# Initial Experience With Stereotactic Radiosurgery for Refractory Trigeminal Neuralgia Using the OXRAY System

**DOI:** 10.7759/cureus.112048

**Published:** 2026-07-04

**Authors:** Shinichiro Mizumatsu, Hiroshi Ryu, Satoshi Yoshikawa, Masaya Ichihara, Fumiya Baba

**Affiliations:** 1 Department of Radiology, Narita Memorial Hospital, Toyohashi, JPN; 2 Department of Neurological Surgery, Narita Memorial Hospital, Toyohashi, JPN; 3 Cerebrospinal Center, Aoyama General Hospital, Toyokawa, JPN; 4 Division of Medical Physics, Narita Memorial Proton Center, Toyohashi, JPN

**Keywords:** elderly patients, flattening filter-free (fff), frameless srs, image-guided radiotherapy (igrt), o-ring type linear accelerator, oxray, pain relief, stereotactic radiosurgery (srs), trigeminal neuralgia, volumetric-modulated arc therapy (vmat)

## Abstract

We report our experience with frameless stereotactic radiosurgery (SRS) using a novel O-ring type linear accelerator (LINAC), the OXRAY system, for refractory trigeminal neuralgia (TN). Three elderly TN patients resistant to pharmacological therapy were included. The head was immobilized with a double-shell mask, and the target was contoured to encompass the entire diameter of the trigeminal nerve at the trigeminal impression. The treatment plan was designed to deliver a maximum dose of 90 Gy in a single fraction using 6 MV flattening filter-free (FFF), volumetric-modulated arc therapy (VMAT). During treatment, image-guided radiotherapy (IGRT) using cone-beam computed tomography (CBCT) was performed a total of five times between each arc. Case 1 was a 74-year-old woman with atypical TN of the left mandibular nerve (V3) and severe numbness, who had undergone three prior microvascular decompressions. SRS was performed with a total irradiation time of 2,492 seconds. Her pain and numbness decreased four months after SRS. At the 16-month follow-up, the Barrow Neurological Institute (BNI) pain score had improved from IV to IIIa, and the facial numbness score had improved from IV to II. The carbamazepine (CBZ) dose was reduced from 500 mg to 100 mg/day. Case 2 was a 95-year-old woman with typical right maxillary nerve (V2) TN. SRS was performed with a total irradiation time of 1,356 seconds. Her pain resolved within a week after SRS, and CBZ was tapered and discontinued over a two-month period. At the 10-month follow-up, the BNI pain score remained at I (improved from IV) without CBZ. Case 3 was an 85-year-old woman with typical right V2 and V3 TN. SRS was performed with a total irradiation time of 1,637 seconds. Her pain resolved the day after SRS, and CBZ was tapered and discontinued over a one-month period. At the five-month follow-up, the BNI pain score remained at I (improved from IV) without CBZ. In all cases, the positional displacement on CBCT was within 1 mm or 1°, and no correction was necessary. No adverse events related to SRS were observed. Frameless FFF-VMAT SRS using the OXRAY system with repeated IGRT demonstrated favorable initial pain relief. This approach may represent a feasible and effective treatment option for elderly patients with refractory TN. Long-term follow-up is necessary to evaluate the incidence of adverse events and recurrence. In the future, the addition of a continuous intrafraction motion monitoring function is expected to reduce radiation exposure, further improve accuracy, and shorten treatment time.

## Introduction

Stereotactic radiosurgery (SRS) is a well-established treatment for refractory trigeminal neuralgia (TN) [1]. Traditionally, the gold standard has been frame-based SRS using the Gamma Knife (GK), which employs isocentric irradiation with multi-circular collimators [2,3]. However, recent advancements in linear accelerator (LINAC) technology have enabled frameless SRS using multi-leaf collimators (MLCs) [1,4]. Since the target in TN is extremely small, high-precision delivery using high-resolution MLCs (e.g., 2.5 mm width) is essential. Furthermore, the adoption of high-dose-rate irradiation, such as flattening filter-free (FFF) beams combined with volumetric-modulated arc therapy (VMAT), contributes to minimizing the risk of patient intrafraction motion by significantly shortening the treatment time [5-7]. In addition, the integration of image-guided radiotherapy (IGRT) with a high-precision double-shell mask has enabled sub-millimeter accuracy, even when using LINAC-based systems [8,9]. The OXRAY® system (Hitachi High-Tech, Tokyo, Japan) is a novel O-ring type LINAC radiotherapy system that commenced clinical operation in June 2024 [10]. This system features 2.5 mm fine MLCs in the central field, integrated with FFF, VMAT, and IGRT to ensure high-precision irradiation. In this report, we describe our initial experience with SRS for TN using the OXRAY system to evaluate its clinical usefulness and identify potential challenges.

## Case presentation

**Figure 1:**
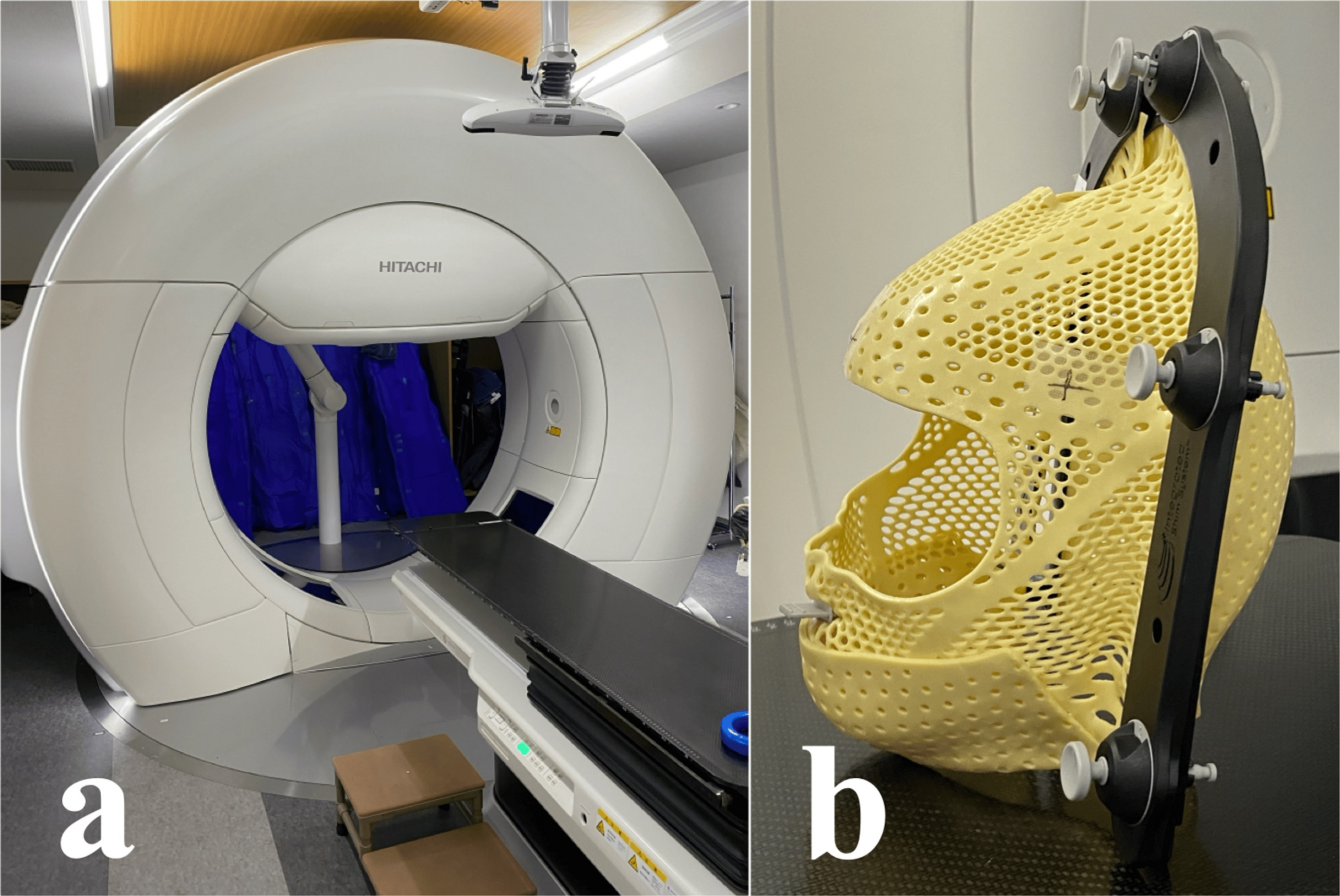
Frameless SRS systems. (a) External appearance of the novel O-ring type linear accelerator, the OXRAY® system (Hitachi High-Tech, Tokyo, Japan). (b) The Encompass™ immobilization system (CQ Medical, Avondale, PA), featuring a thermoplastic double-shell mask. SRS: stereotactic radiosurgery.

Three elderly patients with drug-resistant TN, who were either ineligible for or reluctant to undergo microvascular decompression (MVD), were treated at our facility. These patients sought treatment at our facility due to geographical barriers to existing SRS centers equipped with GK or CyberKnife (CK). All patients underwent SRS using the OXRAY system under a consistent protocol (Figure 1a). For head immobilization, a non-rigid, thermoplastic double-shell mask (Encompass™, CQ Medical, Avondale, PA) was used (Figure 1b).

**Figure 2:**
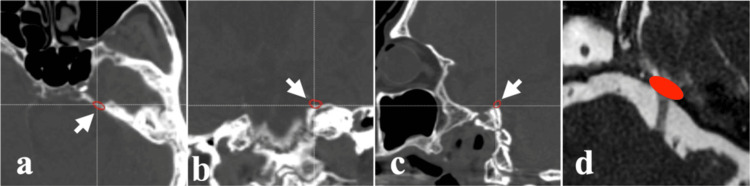
Definition of the treatment target spanning the trigeminal impression on bone-window CT and the planned irradiation area overlaid on T2WI. The treatment target is delineated in red (white arrows). (a) Axial, (b) coronal, and (c) sagittal views. (d) Schematic representation of the planned irradiation area (red) overlaid on axial high-resolution T2WI. CT: computed tomography; T2WI: T2-weighted imaging.

The trigeminal nerve was identified by fusing high-resolution T2-weighted images from the referring institution with non-contrast planning computed tomography (CT) scans (0.625 mm slice thickness). Treatment planning was performed using RayStation software (version 2023B/2024B; RaySearch Laboratories AB, Stockholm, Sweden) with a 1.0 mm dose calculation grid. The target was contoured to encompass the entire diameter of the trigeminal nerve at the trigeminal impression of the petrous bone, near Meckel's cave (Figure 2).

Irradiation was delivered via VMAT using 6 MV FFF beams, with a single-fraction maximum dose of 90 Gy. The O-ring gantry was fixed at 0°, and six arcs were used for the delivery. For each case, all six arcs were set to rotate clockwise, with a starting position of 182° and an ending position of 176°. The dose distribution was optimized to align with the longitudinal axis of the petrous bone. To ensure sub-millimeter accuracy, IGRT using cone-beam CT (CBCT) was performed five times in total, specifically between each arc. The threshold for positional correction was set at 1.0 mm or 1.0° during IGRT. Patient-specific quality assurance was performed through EBT4 Gafchromic film measurements. The treatment planning parameters are summarized in Table 1.

**Table 1:** Dosimetric parameters for the target and organs at risk. Target volumes vary depending on factors such as the width of the trigeminal nerve, the size of the trigeminal impression, and the angle of passage. *D*_max_: point maximum dose; V12Gy: volume receiving at least 12 Gy; V10Gy: volume receiving at least 10 Gy.

Structure	Parameter	Case 1	Case 2	Case 3
Target	Volume (ml)	0.04	0.07	0.06
	*D*_max_ (Gy)	90	90	90
	D95 (Gy)	58.86	59.84	43.01
Brainstem	*D*_max_ (Gy)	14.52	13.60	11.48
	V12Gy (ml)	0.04	0.02	0.00
	V10Gy (ml)	0.01	0.00	0.00
Cochlea	*D*_max_ (Gy)	1.53	0.81	5.58
Internal auditory canal	*D*_max_ (Gy)	1.64	5.20	8.68
Temporal lobe	*D*_max _(Gy)	37.92	33.17	35.79
	V12Gy (ml)	0.28	0.91	0.09
	V10Gy (ml)	0.44	1.27	0.19
Eyeball	*D*_max_ (Gy)	0.70	0.29	3.74

Case 1

**Figure 3:**
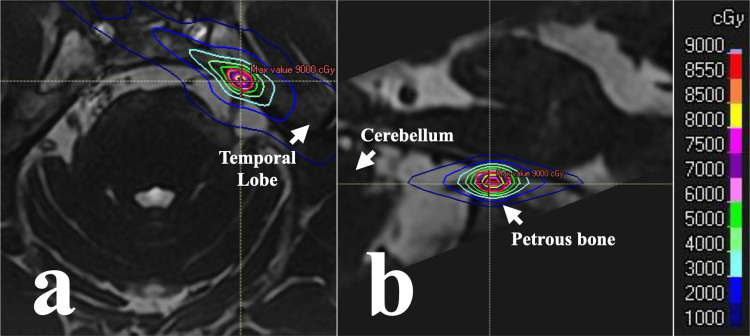
Dose distribution of SRS (Case 1). Isodose lines are shown relative to the maximum dose (100% = 90 Gy) on high-resolution T2WI. (a) Axial view and (b) sagittal view. SRS: stereotactic radiosurgery; T2WI: T2-weighted imaging.

A 74-year-old woman presented with a 12-year history of refractory TN in the left mandibular nerve (V3) territory. She had previously undergone three MVD procedures, various nerve blocks, and multiple pharmacological therapies. She presented with atypical TN; the pain was triggered not only by eating but also by walking and yawning. The pain was so severe that it frequently impaired her mobility for up to 30 minutes. Additionally, she experienced persistent numbness in the left maxillary nerve (V2) and V3 territories. During pain exacerbations, the numbness radiated to the contralateral lower eyelid. Although her pain could be managed with carbamazepine (CBZ) at 500 mg/day, her dosage had to be restricted to 200 mg/day due to severe somnolence. She declined a fourth MVD and was referred to our hospital for SRS. Physical examination revealed no tenderness at the trigeminal nerve exit points. Her baseline Barrow Neurological Institute (BNI) scores for both pain and facial numbness were IV. SRS was performed with a total irradiation time of 2,492 seconds, with a total of 47,781.6 monitor units (MU) delivered (Figure 3).

Following SRS, her symptoms fluctuated for the first four months before stabilizing. At the 16-month follow-up, her pain was well controlled with a reduced CBZ dose of 100 mg/day, and her numbness significantly improved (BNI pain score: IV to IIIa; numbness score: IV to II). No new complications were observed.

Case 2

**Figure 4:**
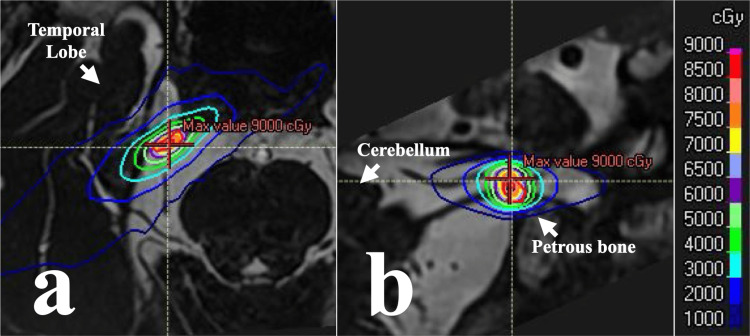
Dose distribution of SRS (Case 2). Isodose lines are shown relative to the maximum dose (100% = 90 Gy) on high-resolution T2WI. (a) Axial view and (b) sagittal view. SRS: stereotactic radiosurgery; T2WI: T2-weighted imaging.

A 95-year-old woman presented with a 16-month history of right-sided gingival pain in the V2 territory. Although her pain initially subsided with 300 mg/day of CBZ, she developed gait disturbance due to dizziness. Subsequent reduction of the CBZ dose to 100 mg/day resolved the dizziness but resulted in inadequate pain control. Given her advanced age, she was considered a poor candidate for MVD and was referred to our hospital for SRS. She exhibited typical paroxysmal pain triggered by gargling or brushing her teeth. Physical examination revealed tenderness over the right infraorbital foramen, with no other neurological deficits. Her baseline BNI pain score was IV, and her facial numbness score was I. SRS was performed with a total irradiation time of 1,356 seconds, with a total of 25,939.2 MU delivered (Figure 4).

Her pain decreased immediately following SRS, and CBZ was gradually tapered and discontinued over a two-month period. At the 10-month follow-up, she remained pain-free without medication (BNI pain score: IV to I), and the tenderness at the right infraorbital foramen resolved. No adverse events were observed.

Case 3

**Figure 5:**
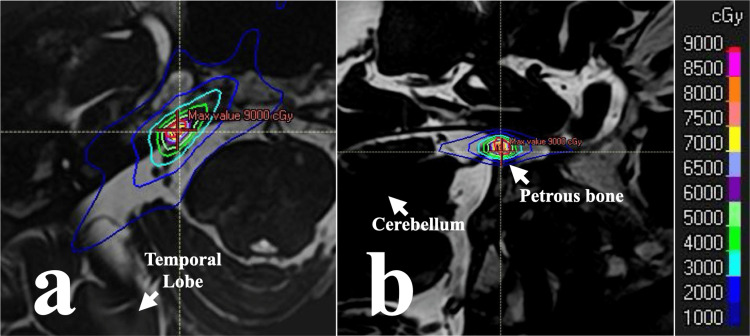
Dose distribution of SRS (Case 3). Isodose lines are shown relative to the maximum dose (100% = 90 Gy) on high-resolution T2WI. (a) Axial view and (b) sagittal view. SRS: stereotactic radiosurgery; T2WI: T2-weighted imaging.

An 85-year-old woman presented with pain in the right V3 territory (the exact duration of her symptoms was unavailable). She had previously received xenon light therapy, lidocaine infusions, and various oral painkillers at a pain clinic, but none were effective. Although she attempted CBZ, doses of 100-200 mg/day caused severe dizziness. The maximum tolerated dose was only 50 mg/day, which failed to provide sufficient pain control. She declined MVD and was referred to our hospital for SRS. The patient presented with typical TN characterized by paroxysmal attacks triggered by eating, leading to decreased oral intake. Physical examination revealed mild tenderness over the right infraorbital foramen and severe tenderness over the mental foramen, with no other neurological deficits. Her baseline BNI pain score was IV, and her facial numbness score was I. SRS was performed with a total irradiation time of 1,637 seconds, with a total of 30,652 MU delivered (Figure 5).

Following SRS, the tenderness over the mental foramen disappeared promptly, while slight tenderness over the infraorbital foramen persisted. Her pain decreased right after the SRS and fully resolved by the following day. CBZ was gradually tapered and discontinued over one month. At the five-month follow-up, she remained pain-free without medication. Tenderness at the right infraorbital and mental foramina had resolved. Her oral intake improved, and she remained in good clinical condition (BNI pain score: IV to I). No adverse events were observed. In all three cases, the positional displacement on CBCT was within 1 mm or 1°, and no correction was necessary (Table 2).

**Table 2:** Inter-arc positional displacements measured during CBCT-based IGRT. The positional correction threshold was set at 1.0 mm or 1.0°. No corrections were required in any case. Mean and maximum values are based on absolute values, as the signs indicate the direction of displacement. CBCT: cone-beam computed tomography; IGRT: image-guided radiotherapy.

Case	Inter-arc	Lateral shift (mm)	Longitudinal shift (mm)	Vertical shift (mm)	Pitch angle (degree)	Roll angle (degree)	Ring angle (degree)
1	Arc 1 to 2	0	-0.4	0.1	0.8	0.2	-0.5
	Arc 2 to 3	-0.2	0.3	-0.3	0.5	0.4	0.5
	Arc 3 to 4	-0.3	0.2	0	0	0.2	0.3
	Arc 4 to 5	-0.3	-0.2	0	0	0.4	0.9
	Arc 5 to 6	-0.1	0.1	0	-0.1	0.3	0.3
	Mean	0.18	0.24	0.08	0.28	0.3	0.5
	Max	0.3	0.4	0.3	0.8	0.4	0.9
2	Arc 1 to 2	0.7	-0.5	-0.6	0.4	-0.2	-0.5
	Arc 2 to 3	0	-0.4	-0.8	0.5	0	-0.1
	Arc 3 to 4	0.2	0	-0.1	-0.4	0.1	0
	Arc 4 to 5	0	-0.3	-0.3	0.4	-0.3	-0.6
	Arc 5 to 6	-0.1	0.1	-0.5	0	-0.4	0
	Mean	0.2	0.26	0.46	0.34	0.2	0.24
	Max	0.7	0.5	0.8	0.5	0.4	0.6
3	Arc 1 to 2	0	0.1	-0.4	0.2	-0.1	0.5
	Arc 2 to 3	0.1	-0.1	-0.2	0.3	0	0.1
	Arc 3 to 4	0	0.4	0.6	-0.6	0.1	0.3
	Arc 4 to 5	-0.2	-0.2	-0.4	0.6	-0.1	0.3
	Arc 5 to 6	0.3	0.1	-0.4	0	-0.1	0.1
	Mean	0.12	0.18	0.4	0.34	0.08	0.26
	Max	0.3	0.4	0.6	0.6	0.1	0.5

## Discussion

The initial pain relief for TN achieved with SRS using the OXRAY system was favorable. Furthermore, Case 1, as an atypical TN, achieved pain relief, improvement in numbness, and a reduced CBZ dosage. This favorable outcome suggests that the SRS protocol may be a highly effective treatment option or a viable salvage therapy, even for patients with intractable atypical TN who have failed multiple MVD and medical therapies. Although the observation period was short, no treatment-related adverse events were observed.

Given the small volume of the TN target, 0.625-mm thin-section CT was utilized for treatment planning to enhance target visibility, contouring accuracy, and detection capability during IGRT. The OXRAY system features a fine MLC with a 2.5-mm central leaf width but does not support circular cones [10]. Therefore, it is challenging to replicate the highly spherical isodose distribution typical of the GK, or the narrow, elongated distribution along the nerve pathway characteristic of the CK [11]. Furthermore, irradiation with the OXRAY MLC inherently yields a high dose gradient in the vertical plane, while producing a more elongated dose distribution in the anterior-posterior and lateral directions. To exploit these dosimetric characteristics, we adopted a strategy of shaping the target volume to cross the nerve transversely within the trigeminal impression near the Gasserian ganglion. Therefore, we intentionally expanded the target volume slightly along the petrous bone and in the vertical direction, but deliberately avoided extending it toward the brainstem or temporal lobe. This intentional contouring resulted in a target volume shape that was wider than the actual width of the trigeminal nerve.

This approach offers three clinical advantages. First, it facilitates target identification and improves IGRT efficiency. Even when visualization of the trigeminal nerve on magnetic resonance imaging is suboptimal or challenging, the anatomical location of the trigeminal impression enables reliable contouring of the target area, as the nerve invariably passes over this bony landmark. Furthermore, the petrous bone serves as a stable, rigid landmark that is clearly visualized even on non-contrast CT, significantly simplifying the registration with CBCT. Second, this approach minimizes the dose to the brainstem, a critical organ at risk. By directing the beam toward the petrous bone, a safe margin from the brainstem is inherently maintained. This strategy mitigates the risk of high-dose spill into the brainstem, even in the event of unexpected patient movement. Third, it provides robustness against setup errors. In frameless SRS, highly spherical isocentric irradiation (typical of the GK) or narrow longitudinal irradiation along the nerve (typical of the CK) carries a substantial risk of a "geographic miss" even with slight lateral displacement. In contrast, creating a transversely broad target area along the petrous bone increases the probability that the nerve remains within the high-dose volume. Importantly, while irradiating a longer segment of the nerve may enhance pain relief, it has been reported to increase the incidence of adverse events, such as sensory disturbances [1,12,13]. In our method, we restrict the elongation of the target volume along the nerve pathway and apply strict dose constraints to the brainstem and temporal lobe during inverse planning. These combined strategies prevent the high-dose volume from extending along the nerve, which is expected to secondarily reduce the risk of such complications. However, long-term follow-up remains essential to fully evaluate these late adverse events. Furthermore, this approach is not limited to the OXRAY system but is generalizable to many conventional LINAC-based SRS systems.

This method also presents several challenges. First, short-segment irradiation may theoretically reduce the long-term pain-relieving effect. To address this potential limitation, we chose 90 Gy, which is the maximum dose commonly used [1]. Although the initial pain relief outcomes were satisfactory, rigorous long-term follow-up is essential to monitor for potential pain recurrence. Second, because the target lies in close proximity to the temporal lobe, the dose to this region is inevitably higher. To prevent radiation necrosis, maintaining volume indices (such as V12Gy and V10Gy) below acceptable thresholds is crucial [14]. In our series, while V12Gy and V10Gy were well within safe limits, the maximum point dose (*D*_max_) to the temporal lobe was consistently elevated (Table 1). In elderly patients, the clinical risk associated with a high *D*_max_ may be mitigated by age-related brain atrophy, which increases the distance between the prescription target and functional brain tissue. Accurately contouring the extensive temporal lobe on CT-MRI fusion images is challenging; therefore, we delineated the nearby dural surface instead. Since *D*_max_ is located on this dural surface, it remains distant from the atrophied brain parenchyma, ensuring relative safety. Consequently, the clinical risk of symptomatic radiation necrosis in our predominantly elderly cohort is expected to be minimal, given the highly localized high-dose region and favorable volume parameters. However, such high-dose irradiation remains a significant concern for younger patients without atrophy, as it carries a lifelong risk of late-onset complications. Finally, all arc irradiations in this study were performed at a 0° O-ring gantry angle. Although a key feature of the OXRAY system is its flexibility to alter the gantry angle [10], determining the optimal non-coplanar angle currently relies on manual selection rather than automated inverse planning. Our preliminary attempts to utilize alternative gantry angles yielded no significant dosimetric advantages, likely due to our limited initial experience and the challenges of manual optimization without automated algorithms. With further clinical experience and advancements in treatment planning software, optimized beam geometry may allow for a further reduction in the dose to the temporal lobe.

One of the major advantages of the OXRAY system is that the integration of VMAT and FFF modes can shorten the irradiation time [5,6,10]. In our series, a notable reduction in irradiation time was achieved from Case 1 to Cases 2 and 3. This improvement is primarily attributed to a learning curve involving the iterative optimization of treatment planning and delivery parameters. This enhanced efficiency not only alleviates the physical burden on elderly patients but also mitigates the risk of intra-fraction patient motion. Moreover, previous literature suggests that higher dose rates may enhance analgesic effects in SRS for TN [15,16]. The OXRAY system provides a high dose rate of 1,200 MU/min in the FFF mode, doubling the 600 MU/min available in the conventional filtered mode [17]. This high dose rate could potentially translate into superior clinical efficacy through enhanced biological effects. Additionally, the FFF mode is expected to minimize unnecessary exposure to surrounding healthy tissues by reducing scattered radiation and leakage [18]. 

In frameless SRS, balancing non-invasiveness with immobilization accuracy is paramount. Double-shell masks, featuring an anterior-posterior sandwich design, provide superior immobilization compared to conventional single-shell masks [8]. This design enhances patient comfort by distributing pressure more evenly and is particularly suited for the high precision required in SRS for TN. Although frameless SRS is less invasive than conventional GK treatment, which requires pin fixation, intrafraction motion monitoring remains a critical requirement. The OXRAY system currently lacks a continuous intrafraction motion monitoring function. To compensate for this limitation, we implemented a rigorous IGRT protocol involving a total of five CBCT scans, performed sequentially between each arc. In all three cases, the positional displacement remained well within our institutional tolerance threshold (1.0 mm or 1.0°). In the future, the addition of a continuous intrafraction motion monitoring function is expected to reduce radiation exposure, further improve accuracy, and shorten treatment time.

Furthermore, the O-ring type LINAC architecture, characterized by a robust circular gantry supported at both ends, exhibits less mechanical wobble and higher geometric precision than conventional C-arm LINACs [19]. This structural stability allows for faster gantry rotation speeds. Because all components are integrated within a cylindrical structure similar to that of a CT scanner, the geometric accuracy of the CBCT is exceptionally high, thereby minimizing registration errors with the planning CT. Additionally, the patient couch remains stationary during irradiation in the OXRAY system, further contributing to highly stable body immobilization.

The recurrence rate after SRS for TN generally increases over time, and repeat SRS may be required in some cases [1,20]. However, due to the dose distribution characteristics of the OXRAY system, avoiding overlapping critical doses during subsequent re-irradiation poses a significant technical challenge compared to GK or CK. Therefore, if further treatment is required, alternative SRS procedures such as GK or CK are typically utilized. Nevertheless, elderly patients may experience difficulty traveling to specialized facilities. In cases with severe symptoms, nearby available interventions, such as nerve blocks, may need to be considered. Consequently, diligent long-term monitoring for both pain recurrence and late complications is essential, particularly given the small sample size and short follow-up period of this initial experience.

## Conclusions

Frameless FFF-VMAT SRS using the OXRAY system with repeated IGRT demonstrated favorable initial pain relief. This approach may represent a feasible and effective treatment option for elderly patients with refractory TN. Long-term follow-up is necessary to evaluate the incidence of adverse events and recurrence. Incorporating a continuous intrafraction motion monitoring function would be highly beneficial to reduce radiation exposure, further improve accuracy, and shorten treatment time.
